# Evaluation of passion fruit mesocarp flour on the paste, dough, and quality characteristics of dried noodles

**DOI:** 10.1002/fsn3.2788

**Published:** 2022-03-01

**Authors:** Xin Ning, Yahan Zhou, Zhen Wang, Xiaodong Zheng, Xiaoli Pan, Zhilin Chen, Qiuping Liu, Wei Du, Xiaohuang Cao, Lei Wang

**Affiliations:** ^1^ College of Chemistry and Food Science Yulin Normal University Yulin China; ^2^ School of Light Industry Beijing Technology and Business University Beijing China; ^3^ Jinan Fruit Research Institute All China Federation of Supply and Marketing Co‐operatives Jinan China; ^4^ School of Physical and Telecommunication Engineering Yulin Normal University Yulin China; ^5^ Guangxi Hong Bang Food Co., Ltd. Yulin China; ^6^ Guangxi Key Laboratory of Agricultural Resources Chemistry and Biotechnology Yulin China; ^7^ Colleges and Universities Key Laboratory for Efficient Use of Agricultural Resources in the Southeast of Guangxi Yulin China

**Keywords:** dietary fiber, dough, dried noodles, fortification, passion fruit mesocarp flour

## Abstract

Reasonable intake of high‐fiber staple food is already one of the most effective measures in fiber deficiency disease prevention and control. Passion fruit mesocarp flour (PFMF), the primary byproduct during passion fruit processing, was utilized to manufacture high‐fiber dried noodles. The presence of PFMF boosted wheat flour gelatinization and retrogradation. The competition for water between PFMF and wheat flour inhibited the formation of the gluten network, which harmed the cooking properties and decreased consumer acceptance of the resulting dried noodles. Nevertheless, PFMF fortification could considerably increase the dietary fiber content of noodles. Especially for noodles with 9% PFMF, the total dietary fiber content was greater than 6%, and they thus could be regarded as a high‐dietary‐fiber food. Generally, the current work demonstrates the feasibility of fabricating PFMF‐enriched dried noodles and their nutritional superiority compared to the corresponding normal product.

## Graphical abstract

Passion fruit mesocarp flour (PFMF) was found to be an excellent source of dietary fiber and polyphenols. It could be utilized as a nutritional fortifier to manufacture high‐fiber dried noodles to satisfy the nutritional requirements of patients who suffer from fiber deficiency diseases.

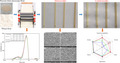

**Highlights**

1. PFMF substitution boosted wheat flour gelatinization and retrogradation.
2. PFMF substitution hindered the formation of the gluten network.
3. The quality of dried noodles was affected by PFMF substitution.
4. The addition of PFMF increased the dietary fiber content of dried noodles remarkably.
5. The fortified dried noodles were an ideal option in regulating dietary structure.

## 1 INTRODUCTION

Worldwide scholars have reached a secure consensus that the high incidence of various chronic metabolic diseases might be closely related to seriously inadequate dietary fiber intake (O'Keefe, 2019; Shah et al., 2020). Fortification with dietary fiber using staple food as a carrier is considered the most direct and effective way to solve the puzzle (Wang et al., 2017). Accordingly, the search for low‐price, high‐quality dietary fiber materials that can pass safety screenings and have far‐ranging sources, as well as an appropriate staple food carrier, has been one of the hotspots in current research.

The current common staple foods include rice, bread, steamed bread, and noodles. Rice is usually eaten directly after cooking; thus, it is inadequate as the carrier. Owing to the action of stalling, the shelf‐life of bread or steamed bread tends to be shorter (Izadi Najafabadi et al., 2014; Zhu, 2016). Even with freezing technology to prolong the shelf‐life, the final quality of related product decreased markedly. Due to their low moisture content, dried noodles have a shelf‐life of up to 1–2 years (Fu, 2008) and therefore are an important branch of the industrial production of wheat flour‐based staple foods, particularly in China, which accounts for approximately 30%–35% of the wheat flour consumption (Chen et al., 2011). In comparison, dried noodles are more suited for manufacturing high‐dietary‐fiber staple foods. Examples of such noodles include shiitake noodles (Wang, Zhao, et al., 2020), dephytinized cereal bran noodles (Levent et al., 2020), chitosan noodles (Zhang et al., 2020), and oat noodles (Nguyen et al., 2020).

Passion fruit is a famous subtropical aromatic fruit. On account of its rich nutrition, pleasant unique flavor, and aroma, passion fruit has been widely used as a desirable ingredient for many formulated foods, especially dairy products and beverages (Ning et al., 2021). Statistics have shown that the annual yield of passion fruit exceeds 0.9 million tons (Corrêa et al., 2016). Peel, accounting for approximately 50%–60% (Vasco‐Correa & Zapata, 2017) of the total fruit weight, especially mesocarp, is the main byproduct inevitably generated during processing. Effective measures should be adopted to dispose of the mesocarp, preferably to convert it into useful products. Dried passion fruit mesocarps contain approximately 15%–20% pectin (Freitas de Oliveira et al., 2016), and its characteristics are similar to those of commercial citrus pectin (Vasco‐Correa & Zapata‐Zapata, 2017); consequently, it is commonly used as feedstock for pectin extraction. However, in practical processes, a substantial amount of residue is generated during extraction, resulting in serious pollution consequences, including large amounts of insoluble dietary fiber quality. Various food byproducts have been extensively identified as potential food ingredients, especially fruit byproducts, due to their profusion of soluble dietary fiber and polyphenol–dietary fiber complex, along with lower content of phytic acid and calories (Ajila et al., 2008). Consequently, a type of high‐dietary‐fiber dried noodle might be manufactured by fortification with passion fruit mesocarp as a natural dietary fiber.

To date, attempts to incorporate passion fruit mesocarps into wheat flour‐based staple foods have scarcely been reported, and the addition of passion fruit mesocarps to dried noodle formulations may cause serious changes between dietary fiber and polyphenols in passion fruit mesocarps and gluten protein in wheat flour, and eventually trigger changes of staple foods’ quality. Thus, the main purpose of this study was to investigate the effects of passion fruit mesocarp incorporation into the paste properties, dough properties, and quality of dried noodles.

## 2 MATERIALS AND METHODS

### 2.1 Materials

High gluten flour with 11.0% protein and food‐grade salt were purchased from the local market and used in dried noodle processing. Naturally matured purple passion fruits were supplied by Guangxi Hongbang Food Co., Ltd. (Yulin, China). Cleaned purple passion fruits were sliced along fruit stalk, and the peels were obtained after removing the pulp. Afterwards, the peels were boiled for 15 min and cooled immediately with ice water. Then, the mesocarp in peels was separated from epicarp using a spoon, and dried to constant weight at 60°C. The 100‐mesh PFMF was obtained from dried mesocarp by grounding and sieving.

To prepare for the blend flour, accurate amounts of PFMF and wheat flour were poured into a pot and manually blended by continuous stirring with a spoon for 3 min. Then, the premixed blend was consecutively passed through an 80‐mesh sieve at least three times, until no significant difference was observed in color homogeneity by naked eyes.

### 2.2 Chemical analysis of PFMF

The moisture was tested by drying samples in an air‐circulating oven at 105°C until constant weight. Kjeldahl method was used to determined protein content according to the Association of Official Analytical Chemists (AOAC) method 960.52 (AOAC, 2006), and the protein content was calculated using the 6.25 factor. Total lipid content was analyzed using the AOAC method 922.06 (AOAC, 2006). After acidic hydrolysis, the crude fat was extracted with ether and petroleum ether. By evaporating the solvent in the supernatant solution with heat, the remaining residual liquid was crude fat. The ash content was measured by a gravimetric method by heating the samples in a muffle furnace at 550°C until light gray ash results, or to constant weight. For dietary fiber contents’ estimation, the samples were digested sequentially by heat‐stable α‐amylase, protease, and amyloglycosidase. The filter residue of enzymatic hydrolysate was regarded as insoluble dietary fiber, the remaining filtrate was precipitated with alcohol and its homologous precipitate was soluble dietary fiber. The total dietary fiber content was calculated as the sum of insoluble dietary fiber and soluble dietary fiber (AOAC, 2006).

### 2.3 Determination of flour pasting properties

Methods in accordance with American Association of Cereal Chemistry (AACC) method 76–21.01 (AACC, 2000), the gelatinization of wheat flour and PFMF substitution on gelatinization of wheat flour were evaluated with a Rapid Visco Analyzer (RVA) (FDV‐E, Shanghai Nirun Intelligent Technology Co., Ltd, China) and 13 min standard test routine (The sample suspensions were held at 50°C for 1 min and heated to 95°C in 3.42 min. The hot paste was held at 95°C for 2.7 min, cooled to 50°C in 3.88 min, and then held at 50°C for 2.0 min. Stirring speed is 960 rpm for 10 s before testing to ensure dispersion of the flour and 160 rpm for the remainder of the test period). The measured parameters were pasting temperature, peak viscosity, setback viscosity, and breakdown viscosity.

### 2.4 Hydration properties of dough

#### 2.4.1 Determination of the gluten index

Gluten content and the gluten index were detected by AACC method 38‐12A (AACC, 2000) using an MJZ‐Gluten index (Hangzhou Daji Photoelectric Instrument Co., Ltd, China). As much as 10.0 g sample was mixed with 4.8 ml of 20 g/L sodium chloride solution for 20 s to a dough, after that, the dough was washed for 5 min with 20 g/L sodium chloride solution, and wet gluten retained was collected. Dry gluten was obtained by drying wet gluten. Wet gluten was put in the gluten index cassette and then centrifuged for 1.0 min at 2000 *g*. Gluten index was quantified as the ratio of wet gluten remaining on sieve to total wet gluten.

#### 2.4.2 Determination of farinograph properties

The farinograph properties of wheat flour and blended flour were carried out using a JFZD‐300 farinograph (Heze Heng tong Laboratory Instrument Ltd., China) using AACC method 54–21 (AACC, 2000). Three hundred grams of flour (%, on 14% moisture flour basis) and appropriate amount of water were kneaded into dough at 30°C, with a maximum consistency of 500 FU (Farinograph units). The stirring resistance during stirring was automatically collected by the computer data acquisition system. The farinograph properties were expressed in terms of water absorption (the amount of water required to reach a dough consistency of 500 FU), dough development time (the time to reach maximum consistency), dough stability time (the time dough consistency remained at 500 FU), and the degree of softening (the consistency difference between height at the peak and the medium line of the torque curve 12 min after development).

#### 2.4.3 Nuclear magnetic resonance (NMR) analysis

A total of 10 g of dough was used for the determination of water mobility using a NM21‐060H‐1 NMR (Shanghai Niumag Electronics Technology Co. Ltd., China). Transverse relaxation (T_2_) was measured using the Carr–Purcell–Meiboom–Gill (CPMG) pulse sequence, then the corresponding CPMG data were fitted using the T_2_‐fit program (Ningbo Jianxin Machinery Co., Ltd., China). The parameters of T2 test included NS = 4, TE = 0.35 ms, TW = 1000 ms, TD = 6994, PRG = 1,NECH = 2000.

### 2.5 Preparation of dried noodles

Dried noodles were prepared, based on the method described in Chinese Standard Method SB/T 10,137 (1993). The basic formulation consisted of 100 g of wheat flour or blend flour, 2 g of salt, and 44% water based on farinograph water absorption. The above raw materials were kneaded into crumbly dough using a dough maker. After 20 min resting, the dough was continuously rolled six times to a 1.0‐mm dough sheet, and finally cut into 3‐mm noodle strands by cutting rollers. These wet noodles were dried in a drying chamber for 10 h under controlled conditions of 40°C and a relative humidity of 70% and then further dried for 10 h at ambient temperature. The dried noodles were preserved in a preservative plastic bag at room temperature until their evaluation.

### 2.6 Microstructure analysis

Dried noodle samples were coated with gold particles. The microstructure of the dried noodle samples was measured by a scanning electron microscope (*SEM*) (6650, OXFORD Instruments, England) at a magnification of 1000‐fold.

### 2.7 Technological properties of dried noodles

#### 2.7.1 Cooking properties

The cooking properties of the dried noodles were evaluated using the standard method as described by LS/T 3212 (2017). Forty dried noodles were randomly selected and cooked in 50 times deionized water. The optimal cooking time was determined by observing how long it took for the white core of the noodles to disappear by squeezing the noodles with two transparent glass slides every 30 s. Another 40 dried noodles were put into 50 times the amount of boiling deionized water until the optimal cooking time. The breaking rate was the proportion of broken cooked noodles to the total number of dried noodles. Water absorption was defined as the weight ratio of noodles before and after cooking. Cooking loss was calculated as the proportion of solid substance lost into the cooking water. Cooked noodles were further analyzed for color, texture, and sensory evaluation.

#### 2.7.2 Color analysis

The surface color management of the dried noodles and cooked noodles was performed using an NS810 portable spectrophotometer. Color parameters, including L* (lightness), a* (redness), and b* (yellowness), were obtained. By the above results, white index (*WI*) was calculated as follows: *WI* = 100‐[(100‐L*)^2^ + a* ^2^ + b* ^2^]^1/2^ (Wang, Ye, Li, et al., 2017).

#### 2.7.3 Texture analysis

A TMS‐PRO texture analyzer equipped with a 100‐N load cell and an acrylic cylinder probe 25.66 mm in diameter (Food Technology Corporation, Virginia, USA) was exploited to evaluate the texture characterization of cooked noodles. Textural parameters were obtained under a texture profile analysis model with the following conditions: a test speed of 0.8 mm/s, compression ratio of 70%, and trigger force of 0.2 N.

### 2.8 Dietary fiber content

The total dietary fiber content of cooked noodles was detected according to the AOAC 991.43 method (AOAC, 2006) by enzymatic–gravimetric analysis.

### 2.9 Sensory evaluation

Thirty trained panelists were recruited to evaluate the color, appearance, texture, aroma, flavor, and overall acceptance of cooked noodles by a 9‐point hedonic scale (1–9, from dislike extremely to like extremely, respectively). This study was approved by the Ethical Review Committee of the Yulin Normal University (Protocol RG 2008.1).

### 2.10 Statistical analysis

All tests were performed in triplicate. The data were evaluated by analysis of variance (ANOVA), and Duncan's test was conducted to compare means. Differences were considered to be significant at *p* < 0.05. SPSS software (version 17.0, SPSS Inc., Chicago, Illinois, USA) was used for statistical computation and analysis.

## 3 RESULTS AND DISCUSSION

### 3.1 Chemical analysis of PFMF

The proximate composition of PFMF is summarized in Table 1. The moisture content, protein content, fat content, and ash content were 5.4%, 6.2%, 4.6%, and 6.4%, respectively. In addition, PFMF contained as high as 75.3% dietary fiber content, of which the soluble dietary fiber was 20.6% and the ratio between soluble dietary fiber and insoluble dietary fiber was 0.38. These values were far beyond those of various grain byproducts. This large amount of soluble dietary fiber indicated that PFMF could be regarded as an interesting ingredient for inclusion in several foods due to the capacity of soluble dietary fiber to retain water and increase postprandial satisfaction, as well as its ability to increase the time needed for nutrient absorption (López‐Vargas et al., 2013).

**TABLE 1:** Proximate composition of passion fruit mesocarp flour

Component	Content
Moisture (%)	5.4 ± 0.1
Total protein (%)	6.2 ± 0.1
Fat (%)	4.6 ± 0.2
Ash (%)	6.4 ± 0.4
Total dietary fiber (%)	75.3 ± 5.6
Soluble dietary fiber (%)	20.6 ± 1.2
Insoluble dietary fiber (%)	54.7 ± 4.5

### 3.2 Effects of PFMF addition on the paste properties of wheat flour

The paste properties of wheat flour with different PFMF additions are shown in Table 2. The peak viscosity (*PV*) of blended flours was linearly positively correlated with the addition of PFMF (*x*), as described by the equation *PV* = 15.53*x *+ 475.34 (*R* ^2^ = 0.92). Water played a role of plasticizer in the pasting of starch. The abundant insoluble dietary fiber in PFMF reduced available water molecules for starch granules owing to its superior capability of binding water. Moreover, dietary fiber could strengthen the hydrogen bonding between dietary fiber and swollen starch, thus causing the increase in viscosity (Wang et al., 2020). The pectin that was leached from PFMF under high temperature conditions should also be addressed. The starch–pectin system could be considered biphasic due to thermodynamic incompatibility between starch and polysaccharides, where pectin interspersed within the starch continuous phase. The continued swelling of starch granules occurring during gelatinization decreased the available water for pectin, thus increasing the concentration of pectin and ultimately the thickening effect of pectin, increasing the viscosity of the starch systems (Dangi et al., 2020).

**TABLE 2:** Effect of passion fruit mesocarp flour addition at various levels on the physicochemical properties of wheat paste and dough

Physicochemical properties		Passion fruit mesocarp flour (%)			
		0	3	6	9
Pasting properties	Pasting temperature (°C)	68.9 ± 0.4^a^	68.6 ± 0.2^a^	66.6 ± 0.3^b^	65.8 ± 0.6^c^
	Peak viscosity (cP)	487.9 ± 2.2^d^	514.1 ± 15.7^c^	546.5 ± 15.9^b^	632.4 ± 32.6^a^
	Breakdown (cP)	285.4 ± 3.2^c^	312.4 ± 2.1^b^	328.7 ± 17.3^b^	357.0 ± 5.6^a^
	Setback (cP)	302.4 ± 2.5^c^	319.5 ± 6.1^b^	327.9 ± 9.5^b^	355.5 ± 8.9^a^
Gluten	Wet gluten yield (%)	31.5 ± 0.2^a^	29.1 ± 0.1 ^b^	24.1 ± 0.2^c^	20.6 ± 0.8^d^
	Dry gluten yield (%)	11.7 ± 0.2^a^	10.6 ± 0.1^b^	8.70 ± 0.01^c^	7.27 ± 0.38^d^
	Gluten index	97.7 ± 1.4^a^	98.6 ± 0.9^a^	98.8 ± 0.4^a^	98.9 ± 0.7^a^
Farinograph	Water absorption (mL/100g)	61.0 ± 0.1^d^	69.6 ± 0.1^c^	77.7 ± 0.2^b^	86.3 ± 0.4^a^
	Dough development time (min)	2.57 ± 0.15^c^	9.73 ± 0.15^a^	9.20 ± 0.36^b^	8.97 ± 0.12^b^
	Dough stability time (min)	14.9 ± 0.2^a^	12.8 ± 0.1^b^	9.80 ± 0.44^c^	5.57 ± 0.23^d^
	Degree of softening (FU)	24.0 ± 2.0^d^	112.7 ± 4.0^c^	122.7 ± 2.1^b^	143.0 ± 1.0^a^

^a–d^Data with different superscript lowercase letters in the same row are significantly different (*p* < 0.05).

The addition of 3% PFMF had a negligible impact on the pasting temperature of wheat flour, while the pasting temperature of wheat flour decreased from 68.9°C to 65.8°C when the addition of PFMF increased from 3% to 9%. This was also probably because of thermodynamic incompatibility between starch and polysaccharides, causing mutual exclusion among the starch phase, protein phase, and polysaccharide phase (Funami et al., 2005). The values of breakdown and setback were used to evaluate the stability of hot and cold paste, respectively. Both were increased with increasing PFMF level. This limited its application in bread, steamed bread, cake, and similar readily staling flour products but had no effect on dried noodles. Similar results were reported by Funami et al. (2005), who found that 1% nonionic polysaccharides, including guar gum, tara gum, and konjac glucomannan, could decrease the pasting temperature of wheat flour and increase the peak viscosity, breakdown, and setback simultaneously.

### 3.3 Effects of PFMF addition on the hydration properties of dough

The wheat flour dough contained 31.5% dry gluten. The wet gluten yields of dough substituted with 3%, 6%, and 9% PFMF were 29.1%, 24.1%, and 20.6%, respectively, which decreased significantly with increasing PFMF substitution. After deducting the dilution factor, the wet gluten yield theoretical values of the three treatments were 30.6%, 29.6%, and 28.7%; consequently, the differences between the theoretical value and experimental value were 1.5%, 5.5%, and 8.1%, which were significantly positively linearly correlated with PFMF substitution (*R* ^2^ = 0.97). Most notably, the difference was greater than the corresponding dilution factor, especially at high substitution. This demonstrated that competition for moisture between wheat flour and PFMF during dough development was the most influential factor, followed by the dilution factor. The changes in dry gluten yield coincided well with those of wet gluten yield. The gluten index was not significantly different from that of the control. The results further demonstrated that competition for moisture and dilution factors were the main affecting factors of PFMF substitution, while at the macro level, the conformational change of gluten induced by PFMF substitution had a negligible impact on gluten strength. The same result was obtained by Wang, Ye, Li, et al. (2017) in oat *β*‐glucan‐fortified wheat dough.

The wheat flour farinograph properties with different PFMF substitutions are presented in Table 2. An apparent increase in water absorption can be observed. This was mainly related to the massive hydroxy group of dietary fiber in PFMF, which features its super water‐absorbing capability. For precisely this reason, PFMF plundered a portion of moisture from wheat flour during dough kneading, thereby decreasing the gluten content and dough strength and eventually leading to a decrease in dough stability time and an increase in the degree of softening. Interestingly, 3% PFMF addition induced a massive increase in dough development time from 2.57 to 9.73 min but decreased slowly thereafter with the continuous increase in PFMF addition. This discrepancy is also related to the competition for water between PFMF and wheat flour, and the greater the amount of PFMF added, the stronger the water‐absorbing capability of the PFMF fraction in dough, and the shorter the equilibrium time needed for kneading dough. The same trend was observed in a study of blended wheat flour and apple pectin (Li et al., 2018).

**FIGURE 1:**
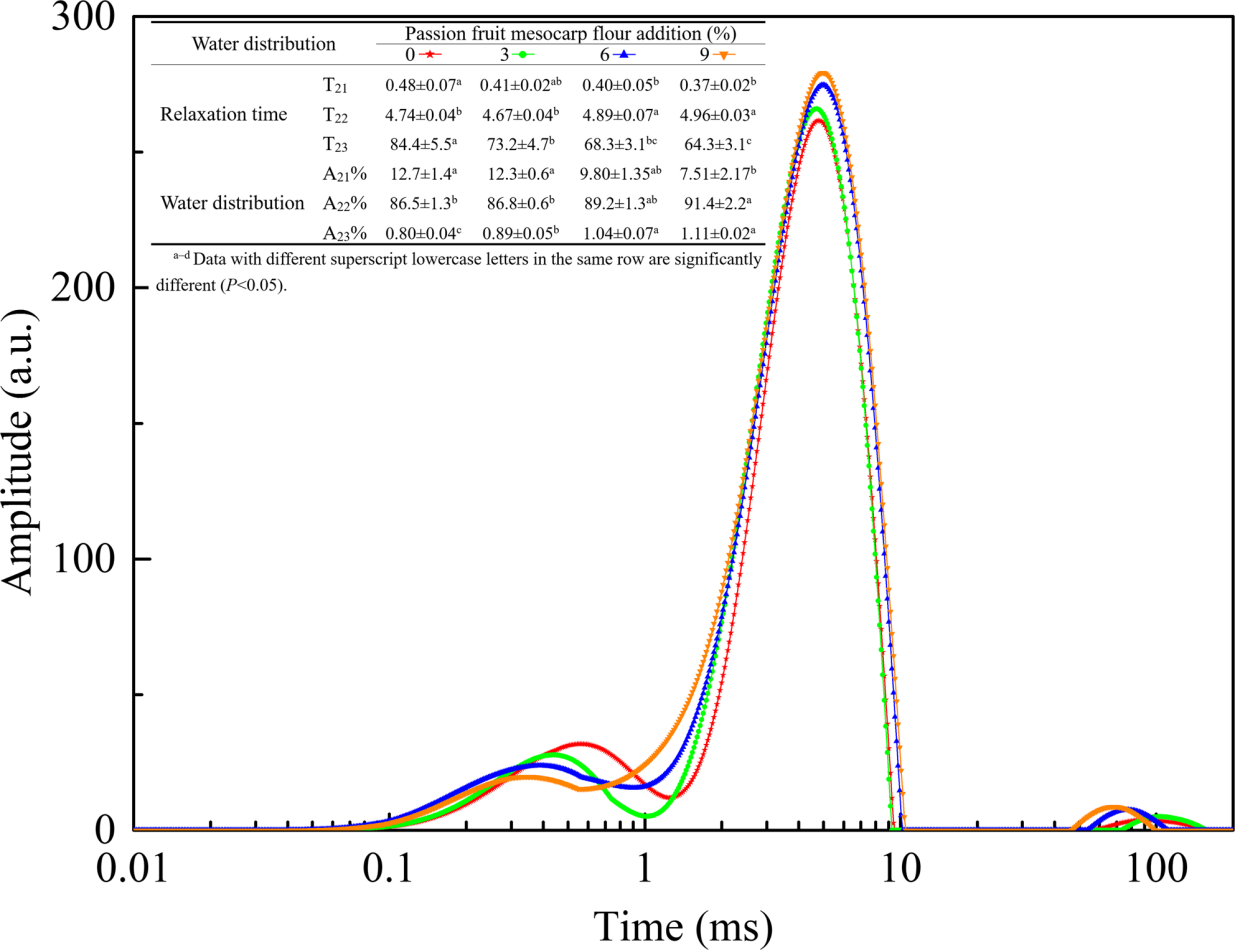
Relaxation T2 distribution of dough enriched with passion fruit mesocarp flour (PFMF). T2 is Spin‐spin relaxation time. ★, control; ●, dough enriched with 3% PFMF; ▲, dough enriched with 6% PFMF; ▼, dough enriched with 9% PFMF

Water content and distribution played a critical role in the processing adaptability of dough and the quality of the corresponding flour products. PFMF fortification markedly changed the water status in the dough. As shown in Figure 1, all samples had three similar CMPG proton populations: T_21_ (0.02–1.10 ms), T_22_ (1.10–10.5 ms), and T_23_ (42.4–214.0 ms), which represent bound water, less tightly bound water, and free water, respectively (Wang, Ye, Li, et al., 2017). Dough fortified with PFMF showed significantly lower T_21_ and T_23_ values. This phenomenon could be attributed to the excellent water‐binding force of PFMF, which lowered the mobility of water in systemic terms.

As the amount of PFMF added increased, A_21_%, regarded as a constituent part of gluten protein, showed a decreasing tendency, while A_22_% and A_23_% increased significantly. The results were inconsistent with those of Zhang et al. (2018), who found that bamboo shoot dietary fiber significantly decreased the content of bound water. This result indicated that PFMF substitution reduced the content of gluten, resulting in reduced bound water, and a fraction of bound water consequently migrated to less tightly bound water and free water. The effects of 3%, 6%, and 9% PFMF dilution on the bound water proportion in dough were 0.38%, 0.76%, and 1.14%, respectively; accordingly, the theoretical values were approximately 12.3%, 11.9%, and 11.5% after removing the dilution effect. Compared with practical test dates, the margins were 0.01%, 2.14%, and 4.05%. This indicated that low incorporation (3%) of PFMF reduced the bound water proportion primarily based on the dilution factor, while for high incorporation (6% and 9%), the competition for water between PFMF and gluten protein was the most major influencing factor, which was significantly higher than the dilution effect.

### 3.4 Effects of PFMF addition on the quality properties of dried noodles

**FIGURE 2:**
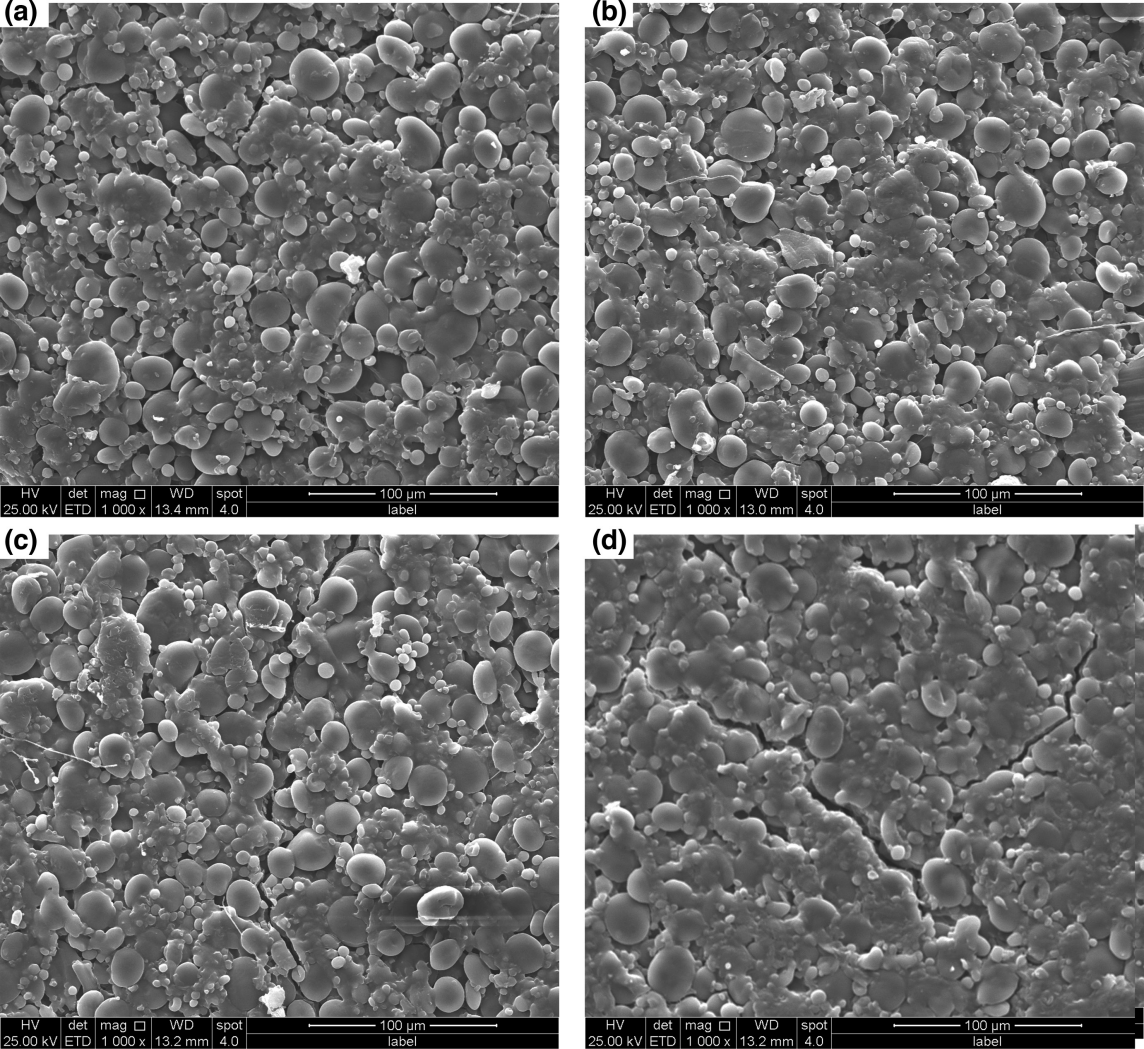
Microstructure of dried noodles enriched with passion fruit mesocarp flour (PFMF). Images represent the following samples: (a) control; (b) dried noodles enriched with 3% PFMF; (c) dried noodles enriched with 6% PFMF; (d) dried noodles enriched with 9% PFMF

The microstructure of dried noodles with and without PFMF incorporation was observed by a scanning electron microscope (*SEM*), as shown in Figure 2. The starch granules with varying sizes were imbedded in nonuniform amorphous gluten protein, and a small fraction of starch granules were exposed to the dried noodle surface. Compared to the control, the sample with 3% PFMF had little effect on the microstructure of dried noodles, while those with 6% and 9% PFMF presented irregular cracks and pits on the surface of dried noodles. It was essentially a manifestation of dough weakening. This allowed the starch granules and dietary fiber to leak more easily from the noodles and was thus adverse to the quality of dried noodles, resulting in higher cooking loss and even breaking rate.

**FIGURE 3:**
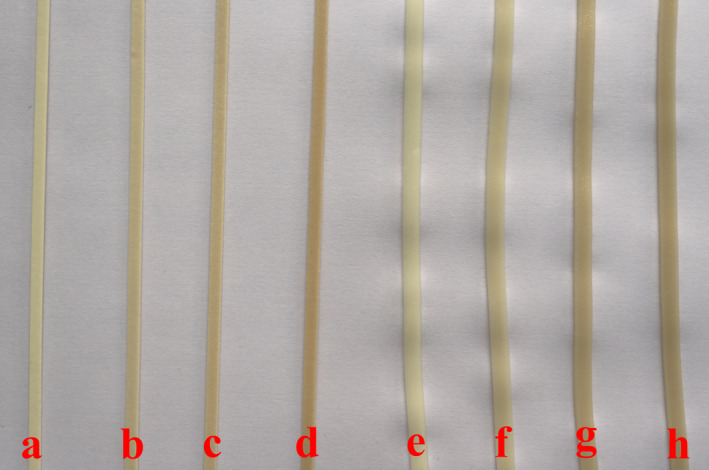
Pictures of dried noodles enriched with passion fruit mesocarp flour (PFMF). Images represent the following samples: (a), control; (b), dried noodles enriched with 3% PFMF; (c) dried noodles enriched with 6% PFMF; (d) dried noodles enriched with 9% PFMF; (e) control cooked dry noodle; (f) cooked dry noodle enriched with 3% PFMF; (g) cooked dry noodle enriched with 6% PFMF; (h) cooked dry noodle enriched with 9% PFMF

As indicators of noodle performance and aesthetic appeal during cooking (Chhikara et al., 2019), the cooking quality parameters are shown in Table 3. The optimal cooking time of dried noodles increased with increasing PFMF addition. All samples maintained a good appearance during cooking (Figure 3). Due to a combination of dilution factors and competition for moisture between PFMF and wheat flour, dough strength was decreased, resulting in lower wrapping capacity of gluten matrix. Consequently, starch granules, dietary fiber, and some soluble materials on the surface of noodles could leak out into the noodle soup. Therefore, compared to the control noodle, the cooking loss of PFMF‐enriched noodles was significantly higher than that of the control noodle. Furthermore, the addition of 3%–9% PFMF caused a slight decrease in water absorption, but there was the same difference among all addition groups. This phenomenon was detrimental to the noodle quality. This result was consistent with the report of Shiau et al. (2020), who reported that noodles enriched with 3%–9% pitaya peel powder had higher cooking loss than the control.

**TABLE 3:** Effect of passion fruit mesocarp flour addition at various levels on the quality of noodle

Quality		Passion fruit mesocarp flour addition			
		0	3	6	9
Cooking properties	Optimal cooking time (min)	7.00 ± 0.00^c^	7.50 ± 0.00^b^	8.00 ± 0.00^a^	8.00 ± 0.00^a^
	Breaking rate (%)	0.00 ± 0.00^a^	0.00 ± 0.00^a^	0.00 ± 0.00^a^	0.00 ± 0.00^a^
	Water absorption (%)	294.5 ± 3.2^a^	255.8 ± 13.6^b^	251.8 ± 10.3^b^	248.9 ± 3.8^b^
	Cooking loss (%)	5.79 ± 0.32^c^	6.17 ± 0.97^bc^	7.29 ± 0.56^ab^	8.04 ± 0.65^a^
Color of dried noodles	L*	84.6 ± 0.3^a^	80.3 ± 0.1^b^	76.3 ± 0.7^c^	72.3 ± 0.6^d^
	a*	1.00 ± 0.02^d^	2.10 ± 0.09^c^	3.36 ± 0.05^b^	4.14 ± 0.10^a^
	b*	10.1 ± 0.1^c^	13.6 ± 0.4^b^	14.9 ± 0.1^a^	15.3 ± 0.2^a^
	*WI*	81.6 ± 0.2^a^	75.9 ± 0.2^b^	71.8 ± 0.6^c^	68.1 ± 0.6^d^
Color of cooked noodles	L*	83.8 ± 0.5^a^	77.6 ± 0.8^b^	73.5 ± 0.3^c^	69.6 ± 0.2^d^
	a*	1.50 ± 0.05^d^	2.53 ± 0.15^c^	3.64 ± 0.07^b^	4.47 ± 0.11^a^
	b*	10.3 ± 0.1^d^	11.5 ± 0.4^c^	13.0 ± 0.2^b^	14.6 ± 0.2^a^
	*WI*	80.8 ± 0.5^a^	74.7 ± 0.3^b^	70.3 ± 0.3^c^	66.0 ± 0.3^d^
Texture	Hardness (*N*)	56.3 ± 2.8^c^	63.6 ± 1.4^b^	67.2 ± 2.2^a^	63.4 ± 1.3^b^
	Adhesiveness (mJ)	0.95 ± 0.17^b^	1.39 ± 0.12^a^	1.53 ± 0.19^a^	1.57 ± 0.26^a^
	Cohesiveness	0.57 ± 0.04^a^	0.47 ± 0.03^b^	0.40 ± 0.03^c^	0.35 ± 0.01^d^
	Springiness (mm)	0.87 ± 0.04^a^	0.88 ± 0.02^a^	0.91 ± 0.03^a^	0.89 ± 0.03^a^
	Gumminess (*N*)	32.8 ± 3.0^a^	28.9 ± 1.3^b^	27.1 ± 2.1^b^	22.0 ± 1.2^c^
	Chewiness (mJ)	28.5 ± 3.4^a^	25.5 ± 1.4^b^	24.6 ± 2.8^b^	19.7 ± 1.6^c^
Dietary fiber and polyphenols	Soluble dietary fiber (%)	0.77 ± 0.08^d^	1.50 ± 0.08^c^	1.78 ± 0.16^b^	2.46 ± 0.20^a^
	Insoluble dietary fiber (%)	1.44 ± 0.09^d^	2.11 ± 0.06^c^	2.65 ± 0.17^b^	3.57 ± 0.22^a^
	Total dietary fiber (%)	2.22 ± 0.16^d^	3.61 ± 0.13^c^	4.44 ± 0.29^b^	6.03 ± 0.21^a^

^a–d^Data with different superscript lowercase letters in the same row are significantly different (*p* < 0.05).

The color profiles in terms of the L*, a*, b*, and *WI* values of the cooked noodles are shown in Table 3. The a* value and b* value of both dried and cooked noodles increased significantly, and the L* value and *WI* value decreased significantly with increasing PFMF level. The results were attributed to the inherent color of PFMF. According to industry standard LS/T 3304–2017 (Dried noodles, 2017), the sole requirement for the color of dried noodles was that the color should be uniform. Therefore, the color changes, which were caused by the addition of PFMF, were permitted and should not be summed up as quality defects.

Texture properties were major attributes influencing the mouth feel of dried noodles. As seen in Table 3, the hardness first increased and then decreased with the increasing addition of PFMF, and the maximum was obtained with the addition of 6% PFMF. Increasing the PFMF addition level decreased the cohesiveness, gumminess, and chewiness while increasing adhesiveness in a dose‐dependent manner. The addition of 3%–9% PFMF had no visible effect on the springiness. The gluten content, dough strength, and components (especially the dietary fiber content) were the primary factors influencing the texture of the dried noodles, which were appreciably affected by PFMF, especially for the highest quantity additions. Similarly, Zhu and Li (2019) found that noodles fortified with ground linseed impair the texture properties.

Passion fruit mesocarp flour was a rich source of dietary fiber; consequently, the PFMF‐enriched noodles showed significantly high volumes of soluble dietary fiber, insoluble dietary fiber, and total phenolics. In particular, in the 9% group, the total dietary fiber content was greater than 6%, and thus it could be regarded as a high‐dietary‐fiber food. It is of great significance for people who chronically consume refined staple food and offers important health benefits for human health sustainability.

### 3.5 Effects of PFMF addition on the consumer acceptability of dried noodles

**FIGURE 4:**
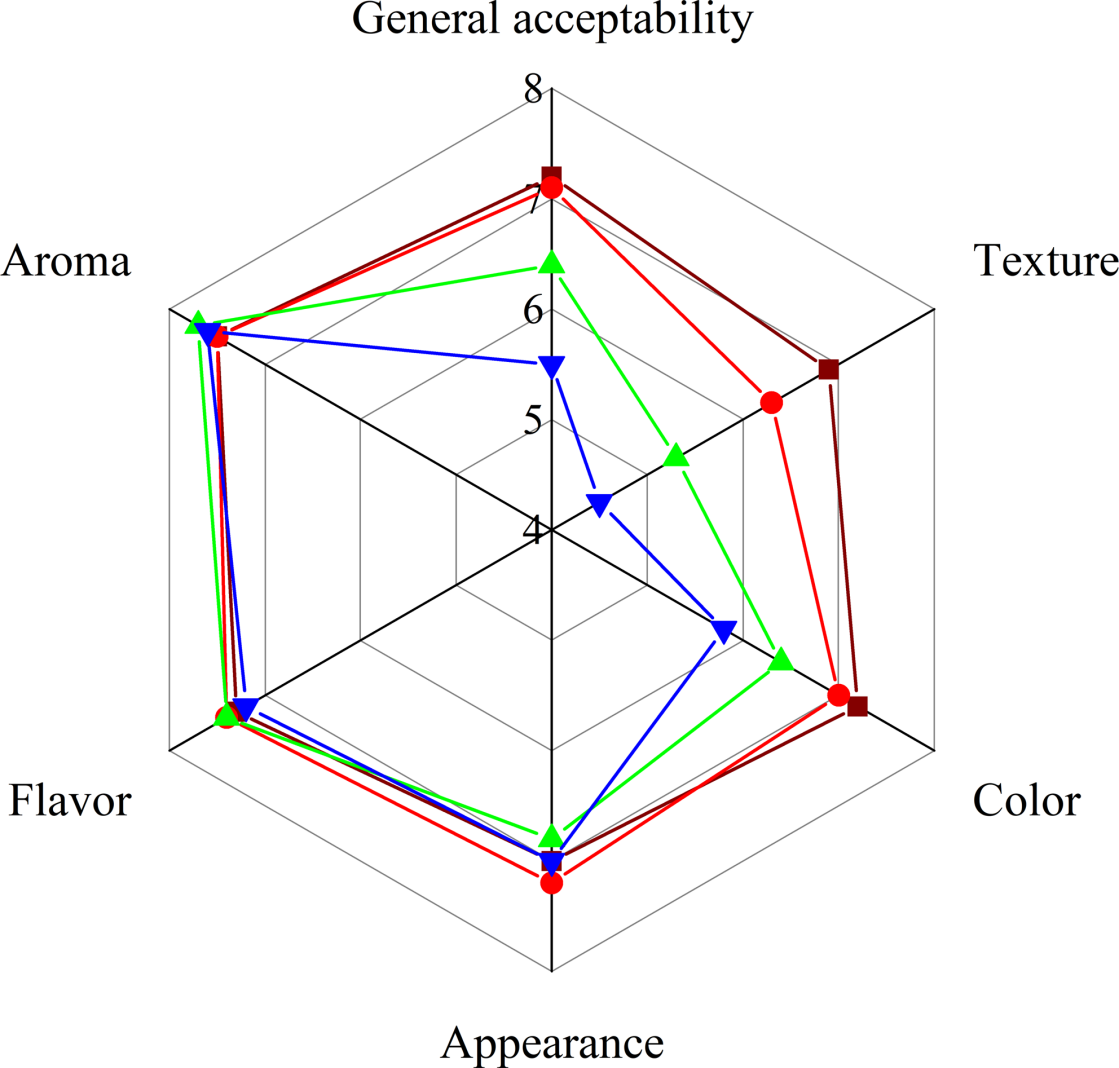
Radar chart for the comparison of the sensory attributes of the control and cooked dry noodles enriched with passion fruit mesocarp flour (PFMF). ■, control; ●, dried noodles enriched with 3% PFMF; ▲, dried noodles enriched with 6% PFMF; ▼, dried noodles enriched with 9% PFMF

Obviously, PFMF had no significant effect on the appearance, aroma, and flavor of the dried noodles (Figure 3 and 4). The industry standard LS/T 3304–2017 (Dried noodles, 2017) had no special requirements for the color of dried noodles, but most consumers prefer noodles with bright colors, thereby reducing the color score and general acceptability score of PFMF‐fortified dried noodles. In addition, a significant decrease in texture was observed when PFMF was added 6%–9%, which was less chewy compared to the control group. Thereby, in a consumer acceptability sense, the amount of PFMF should not exceed 6.0%.

## 4 CONCLUSIONS

Passion fruit mesocarp flour was found to be an excellent source of dietary fiber and polyphenols. It could be utilized as a nutritional fortifier to manufacture high‐fiber dried noodles to satisfy the nutritional requirements of patients who suffer from fiber deficiency diseases, especially dried noodle substitution with 9% PFMF. Notably, high substitution levels of PFMF disturbed the formation of the gluten network and thus marred the cooking properties and consumer acceptability of dried noodles. Hence, it was feasible to manufacture dried noodles with superior nutrition and acceptable taste by incorporating 6% PFMF. Consequently, further studies should focus on ameliorating or overcoming the adverse effects.

## CONFLICT OF INTEREST

The authors declare no conflicts of interest.

## AUTHOR CONTRIBUTION

**Xin Ning:** Investigation (equal); Methodology (equal); Writing – original draft (lead); Writing – review & editing (equal). **Yahan Zhou:** Data curation (equal); Investigation (equal); Methodology (equal). **Zhen Wang:** Data curation (equal); Investigation (equal); Methodology (equal); Writing – original draft (equal). **Xiaodong Zheng:** Methodology (equal); Project administration (equal); Resources (equal); Supervision (equal). **Xiaoli Pan:** Project administration (equal); Software (equal); Visualization (equal). **Zhilin Chen:** Formal analysis (equal); Visualization (equal); Writing – original draft (equal). **Qiuping Liu:** Methodology (equal); Validation (equal). **Wei Du:** Resources (equal); Supervision (equal); Validation (equal). **Xiaohuang Cao:** Conceptualization (equal); Investigation (equal); Methodology (equal); Project administration (equal); Writing – review & editing (equal). **Lei Wang:** Conceptualization (equal); Funding acquisition (lead); Investigation (equal); Methodology (lead); Project administration (equal); Writing – review & editing (equal).
